# A Novel Liquid Chromatography–Tandem Mass Spectrometry Method to Quantify Tryptophan and Its Major Metabolites in Serum to Support Biomarker Studies in Patients with Cancer Undergoing Immunotherapy

**DOI:** 10.3390/molecules30010121

**Published:** 2024-12-31

**Authors:** Anna Siemiątkowska, Barbara Kuźnar-Kamińska, Katarzyna Kosicka-Noworzyń, Kamila Nowaczewska, Hanna Winiarska, Dominika Popiołek, Filip Kamiński, Franciszek K. Główka

**Affiliations:** 1 Department of Physical Pharmacy and Pharmacokinetics, Poznan University of Medical Sciences, 3 Rokietnicka Street, 60-806 Poznań, Poland; kasiakosicka@ump.edu.pl (K.K.-N.); glowka@ump.edu.pl (F.K.G.); 2 Department of Pulmonology, Allergology and Respiratory Oncology, Poznan University of Medical Sciences, 84 Szamarzewskiego Street, 60-569 Poznań, Poland; kaminska@ump.edu.pl (B.K.-K.); winiarskahanna@ump.edu.pl (H.W.);

**Keywords:** cross-signal contribution, cross-interferences, cross-talk, standard impurity, similar MRMs, poor analyte stability

## Abstract

Tryptophan (TRP) is an essential amino acid crucial for the production of many bioactive compounds. Disturbances in TRP metabolism have been revealed in various diseases, many of which are closely related to the immune system. In recent years, we have focused on finding blood-based biomarkers of successful immunotherapy in cancer. Thus, we aimed to develop a robust liquid chromatography–tandem mass spectrometry (LC-MS/MS) method for TRP and its metabolites that could be used in biomarker studies. Although analyzing TRP derivatives in biological matrices is not a new topic, we encountered multiple challenges during method development. One of them was the phenomenon of cross-interferences between the analyzed molecules, which has not been explored in most published papers. We noticed that injecting a pure single-compound solution often generated a signal in the other compounds’ MS/MS channels. Specifically, TRP generated unexpected peaks in the channel for kynurenine, kynurenic acid, and xanthurenic acid, while kynurenine generated peaks in the channel for kynurenic acid. We also recorded a mutual cross-talk between kynurenine and isotope-labeled TRP. Different origins of the observed cross-signal contribution were proposed. This paper draws attention to investigating cross-interferences in LC-MS/MS, especially when structurally related compounds will be analyzed. Despite all the challenges, the method was successfully validated according to international guidelines (EMA/ICH), and its applicability was confirmed in a pilot study including 20 patients with lung cancer undergoing chemoimmunotherapy.

## 1. Introduction

**Figure 1:**
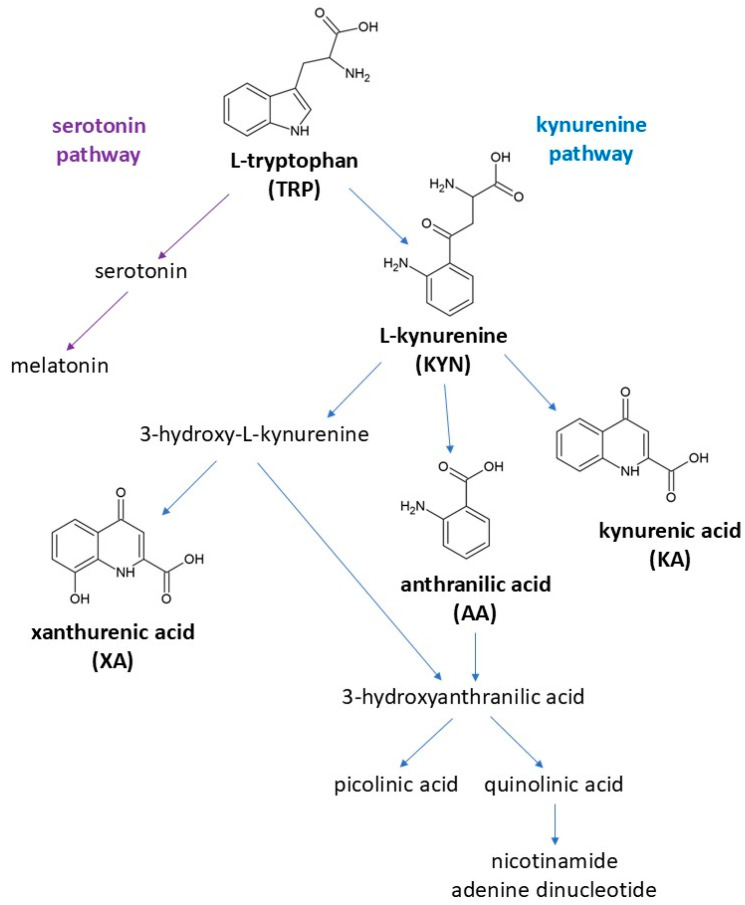
Tryptophan metabolism along the serotonin and kynurenine pathways.

Tryptophan (TRP) is an amino acid crucial for the production of many bioactive compounds, such as serotonin and melatonin [1]. In humans, TRP is not synthesized in the body and must be provided through the diet [2]. TRP undergoes metabolism along two main routes, the serotonin and the kynurenine pathways, with the latter accounting for 95% of its degradation [1,2]. The major TRP metabolite is kynurenine (KYN), which further transforms into multiple downstream products, e.g., anthranilic acid (AA), kynurenic acid (KA), and xanthurenic acid (XA) [3] (Figure 1).

Disturbances in TRP metabolism have been revealed in various diseases, many of which are closely related to the immune system and inflammation [4]. For this reason, the evaluation of TRP metabolism has recently received closer attention in patients undergoing therapies that support the immune system [5,6,7]. In recent years, our group has focused on finding blood-based biomarkers of successful immunotherapy with anti-PD-1/PD-L1 antibodies [8,9]. Thus, we aimed to develop and validate a robust liquid chromatography–tandem mass spectrometry (LC-MS/MS) method for TRP and its major metabolites to apply in ongoing biomarker studies.

TRP metabolites have been analyzed in biological matrices, and many methods have been published, usually based on the LC-MS/MS (Table 1). Despite there being well-established bioanalytics for TRP measurement, the simultaneous analysis of TRP and its metabolites may be challenging for several reasons, e.g., a lack of authentic blank matrices for the calibrators due to the endogenous nature of the analytes. The TRP levels in biological matrices are also much higher compared to metabolites (e.g., [10,11,12]), which may affect the method’s sensitivity (in the case of metabolites) or saturate the signal (for TRP) [13]. These challenges often result in the determination of TRP in separate analytical runs (e.g., [10,11,14,15]), which is undesired in routine practice. Moreover, TRP metabolites differ in physicochemical properties (e.g., hydrophobicity, pKas, and solubility), which may cause problems with chromatographic separation or ionization or cause incomplete recovery [16,17].

Here, we emphasize another aspect to consider when analyzing TRP derivatives by LC-MS/MS: during method development, we observed various cross-interferences between the analyzed molecules, which have not been explored in most published papers. We noticed that injecting a pure single-compound solution often generated a signal in the other compounds’ MS/MS channels, which could have affected the method’s accuracy if it had not been recognized. Any cross-interferences were rarely mentioned in the TRP methods [11,13,18,19], while—to the best of our knowledge—a separate cross-signal contribution experiment was not reported in any of the studies. Thus, this paper draws attention to investigating cross-interferences in LC-MS/MS, especially when structurally related compounds will be analyzed. Despite all the challenges, the proposed method was successfully validated according to international guidelines [20], and its applicability was confirmed in a pilot study in patients undergoing chemoimmunotherapy.

**Table 1:** Selected LC-MS/MS methods for the determination of TRP and its metabolites in human blood (plasma or serum) published over the past 10 years.

Authors (ref.)	Analytes ^a^ (Corresponding IS)	Calibrators	Sample Clean-Up	Matrix Volume	Comment
Choi et al., 2016 [21]	TRP (TRP-D_3_), KYN (KA-D_5_), AA (KA-D_5_), KA (KA-D_5_), and others	in 40 mg/L BSA	PPT with 0.1% FA and ACN	50 µL	no stability and ME tests on authentic matrix
Hu et al., 2017 [15]	TRP (^13^C_4_,^15^N-KYN), KYN (^13^C_4_,^15^N-KYN), and KA (KA-D_5_)	in charcoal-stripped serum	PPT with MeOH	100 µL	no stability and ME tests on authentic matrix
Karakawa et al., 2019 [19]	TRP (^13^C_11_,^15^N_2_-TRP), KYN (^13^C_4_,^15^N-KYN), AA (KA-D_5_), KA (KA-D_5_), XA (XA-D_4_), and others	working solutions used as calibrators	PPT with ACN	60 µL	no stability tests and ME experiment
Whiley et al., 2019 [13]	TRP (TRP-D_5_), KYN (KYN-D_4_), KA (KA-D_5_), XA (XA-D_4_), and others	in water with citric acid	PPT with MeOH and AmmF + SPE ^b^	30 µL	no stability tests on authentic matrix
Trepci et al., 2020 [22]	TRP (TRP-D_3_), KYN (KYN-D_4_), KA (KA-D_5_), XA (XA-D_4_), and others	in water	PPT with ZnSO_4_ and MeOH	30 µL	stability tested on authentic matrix
Tömösi et al., 2020 [16]	TRP (TRP-D_5_), KYN (KYN-D_4_), AA (KA-D_5_), KA (KA-D_5_), XA (XA-D_4_), and others	in charcoal-stripped serum	PPT with 0.1% FA and acetone/MeOH (1:1)	100 µL	no stability tests and ME experiment
Sadok et al., 2021 [11]	TRP (3-NT), KYN (3-NT), KA (3-NT), XA (3-NT), and others	in charcoal-stripped serum	PPT with 15% TCA	95 µL	no stability and ME tests on authentic matrix
Nadour et al., 2022 [14]	TRP (TRP-D_5_), KYN (KYN-D_4_), KA (KA-D_5_), XA (XA-D_4_), and others	in 0.1% FA	PPT with 0.1% FA and MeOH	50 µL	stability tested on authentic matrix
Desmons et al., 2022 [12]	TRP (TRP-D_8_), KYN (TRP-D_8_), KA (15N-AA), XA (TRP-D_8_), and others	in lyophilized blank serum	PPT with MeOH	50 µL	lyophilized blank serum used for stability tests; no ME test for serum
Wang et al., 2023 [10]	TRP (TRP-D_5_), KYN (TRP-D_5_), KA (KA-D_5_), XA (XA-D_4_), and others	in charcoal-stripped 4% BSA in PBS	SPE ^c^	10 µL	stability and ME tests only for charcoal-stripped BSA

^a^ TRP metabolites analyzed also in the presented LC-MS/MS method were mentioned by name; ^b^ phospholipid removal by PHREE SPE plates; ^c^ SPE with Oasis HLB cartridges. *Abbreviations*: 3-NT, 3-nitrotyrosine; AA, anthranilic acid; ACN, acetonitrile; AmmF, ammonium formate; BSA, bovine serum albumin; FA, formic acid; IS, internal standard; KA, kynurenic acid; KYN, kynurenine; ME, matrix effect; MeOH, methanol; PBS, phosphate-buffered saline; PPT, protein precipitation; SPE, solid-phase extraction; TCA, trichloroacetic acid; TRP, tryptophan; XA, xanthurenic acid.

## 2. Results

### 2.1. Cross-Signal Contribution Experiment

The results from the cross-signal contribution experiment, including the extent of cross-interference, are illustrated in Table 2. The mass spectrometer detected signals in the multiple reaction monitoring (MRM) channels of specific compounds in the following situations:

- The signal for TRP appeared only after injecting pure TRP (retention time, RT = 2.3 min);
- The signal for KYN appeared after injecting pure KYN (RT = 1.75 min), but also pure TRP (RT = 1.75 min, **interference I**) and pure TRP-D_5_ (RT = 2.25 min, **interference II**);
- The signal for AA was detected only after injecting pure AA (RT = 4.3 min);
- The signal for KA appeared after injecting pure KA (RT = 6.1 min), but also pure TRP (RT = 2.3 min, **interference III**) and pure KYN (RT = 6.1 min, **interference IV**);
- The signal for XA was detected after injecting pure XA (RT = 6.15 min), but also pure TRP (RT = 2.3 min, **interference V**);
- The signal for 3-nitrotyrosine (3-NT) was detected only after injecting pure 3-NT (RT = 1.7 min);
- The signal for TRP-D_5_ appeared after injecting pure TRP-D_5_ (RT = 2.25 min), but also pure KYN (RT = 1.75 min, **interference VI**).

### 2.2. Validation Results

#### 2.2.1. Selectivity and Carry-Over

The exemplary chromatograms of the blank and lower limit of quantitation (LLOQ) samples are presented in Supplementary Figure S1. The figure also shows a chromatogram for serum obtained from a patient dosed with chemoimmunotherapy. The detailed results from the selectivity experiment can be found in Table S1. For neither compound, the observed peak areas in the blank samples exceeded the acceptable level of interference. No carry-over was noted.

#### 2.2.2. Linearity, Accuracy, Precision, and Matrix Effect

The method was linear in the range of 2–125 µM for TRP, 0.16–10 µM for KYN, and 8–500 nM for AA, KA, and XA. The power function was preferred over the linear regression, providing better accuracy and lower matrix effects. The method’s accuracy ranged from 91.8 to 107.5%, precision ranged from 1.8 to 8.9% (Table S2), and the matrix effects ranged from 93.3 to 104.5% (Table 3). The exemplary calibration curves and their equations are presented in Figure S2.

#### 2.2.3. Stability

The results of the stability tests are presented in Table S3. The methanolic working solutions were stable for at least 4 h at 20 °C, with four cycles of freezing and 5 weeks at −80 °C. A visible decrease in concentrations was revealed for XA in the charcoal-stripped serum after three cycles of freezing and thawing. All other results were acceptable.

### 2.3. Clinical Application

**Figure 2:**
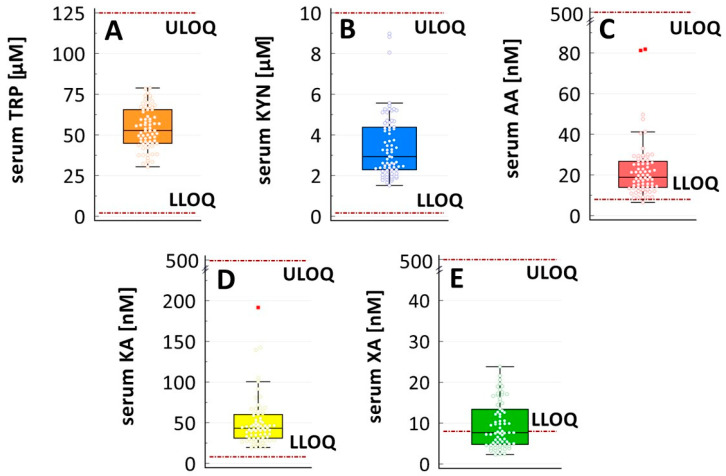
Concentrations of (**A**) tryptophan, (**B**) kynurenine, (**C**) anthranilic acid, (**D**) kynurenic acid, and (**E**) xanthurenic acid in 20 patients with lung cancer undergoing chemoimmunotherapy with pembrolizumab. Serum samples (*n* = 71) were collected at baseline and during treatment. Data are presented as medians along with interquartile ranges (boxes), ranges without outliers (whiskers), and extreme outliers (red-filled squares). Method’s calibration ranges are highlighted with red horizontal lines (LLOQ, lower limit of quantitation; ULOQ, upper limit of quantitation).

In total, 71 serum samples were analyzed. The validated LC-MS/MS method captured all TRP, KYN, and KA concentrations and most (98.6%) AA concentrations. However, the XA levels were below the LLOQ in half of the samples (52.1%). The determined serum concentrations were 52.7 (44.9–65.6) µM for TRP, 2.94 (2.29–4.38) µM for KYN, 18.86 (13.91–26.69) nM for AA, 43.2 (31.04–60.14) nM for KA, and 7.70 (4.80–13.39) nM for XA [medians (interquartile ranges); Figure 2]. Following the approach sometimes utilized in biomarker studies [23], all determined concentrations were presented, including those below the LLOQ threshold.

## 3. Discussion

### 3.1. Cross-Signal Contribution

Due to its selectivity, sensitivity, and high throughput, mass spectrometry is considered a gold standard in bioanalysis [24]. However, special attention must be paid when analyzing structurally related compounds (e.g., in metabolomics [25,26] or lipidomics [27]) as they might share similar fragments and interfere with each other. In the developed assay, we observed explicit cross-interferences in the mass spectrometer responses of four out of seven compounds, i.e., KYN, KA, XA, and TRP-D_5_ (interferences I–VI; Table 2). Thus, it was necessary to troubleshoot the issue and investigate its impact on the quantitative analysis.

Starting from interferences I and IV, we concluded that minor contamination of the reference standards (the TRP standard with KYN and the KYN standard with KA) was probably responsible for the observed peaks as the interferences matched the retention times of the monitored molecules. This contamination may have occurred during the production process as all compounds were structurally related (Figure 1). To exclude the accidental contamination of stock and working solutions, we conducted the cross-signal contribution experiment twice (after replacing all solutions), and the results were comparable. The third experiment, performed in charcoal-stripped serum, showed that the impurities did not exceed 20% of the KYN and KA’s signals at the LLOQ level. Thus, we found these impurities acceptable.

The potential contaminants and degradation products of TRP and KYN have been reviewed by Bellmaine et al. [28], and our observations are in line with those data. Of note, due to large discrepancies in the concentrations of TRP and its metabolites in biological matrices (and between TRP metabolites themselves, e.g., Figure 2), even reference standards with high purity (≥98%) may not guarantee sufficient accuracy of the method (traces of KYN in the TRP’s standard may affect the LLOQ for KYN, while traces of KA in the KYN’s standard may affect the LLOQ for KA). Thus, the level of cross-contamination should always be assessed before a quantitative analysis of these related compounds. Unfortunately, this is not common in published papers.

Interferences II, III, V, and VI most likely resulted from MRMs or fragment patterns that were too similar in the analyzed molecules, as interferences matched the retention times of the injected compounds. The signal cross-talk may occur in LC-MS/MS if compounds have similar precursor and product ions [29]. In our case, there was only a 1 *m*/*z* difference between the precursor ions of KYN and TRP-D_5_ (208.8 vs. 209.8), and their product ions were almost identical (191.9 vs. 192.0). Thus, we concluded that the mass analyzer could not differentiate between KYN and TRP-D_5_ (interferences II and VI). The cross-signal contribution between those molecules was previously mentioned by others [18]. Interestingly, we observed the mutual cross-talk between KYN and TRP-D_5_ also for transitions 208.8 > 145.9 and 209.8 > 150.0 (Figure S3), which seemed to differ enough to be distinguished by the mass spectrometer (4 *m*/*z* difference in the product ions). The phenomenon could be explained by the specific fragment pattern of TRP-D_5_, for which the product ion scan revealed numerous ions at *m*/*z* of 145–151 (Figure S4).

**Figure 3:**
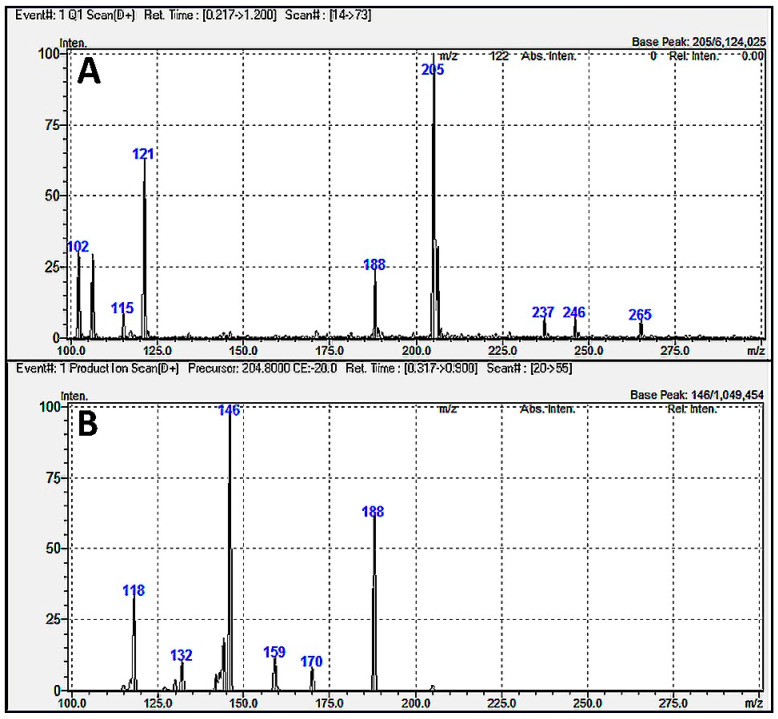
Results of (**A**) Q1 scan and (**B**) product ion scan for tryptophan.

We also suspected a similar fragment pattern as a reason for the cross-signal contribution between TRP and XA (interference V) and TRP and KA (interference III), though their case was less obvious. Namely, TRP and XA showed similar precursor ions (204.8 and 206.0), but their product ions seemed different (145.9 and 159.9). Conversely, TRP and KA had completely different precursor ions (204.8 vs. 189.8) but similar product ions (145.9 and 144.0). In both cases, the cross-signal contribution should not occur. However, the TRP Q1 mass spectrum (Figure 3A) revealed multiple species, including an ion at an *m*/*z* ratio of 188 (i.e., very close to the KA’s precursor ion of 189.8). Thus, an in-source TRP fragmentation could explain why TRP (with a possible transition of 188.0 > 145.9) contributed to the signal of KA (with a monitored transition of 189.8 > 144.0). Furthermore, the TRP product ion scan (Figure 3B) showed a peak at an *m*/*z* value of 159 (while the XA’s product ion was 159.9). Thus, a similar MRM transition was also most likely responsible for the cross-signal contribution between TRP (with a possible transition of 204.8 > 159.0) and XA (with a monitored transition of 206.0 > 159.9).

The cross-signal contribution between TRP and XA was previously noticed in the work of Karakawa et al. [19] and Nadour et al. [14], though not specifically mentioned by the authors: a visible peak appeared in the XA channel of 206 > 160 at the retention time of TRP. In turn, the 205 > 159 transition for TRP was monitored by Sadok et al. [11], who highlighted the possibility of cross-talk between TRP and XA. Whiley et al. [13] showed that TRP contributed to the XA’s signal also in the 206 > 132 channel, which agreed with our observation that one of the TRP fragments had an *m*/*z* ratio of 132 (Figure 3B). According to our knowledge, no study has mentioned (or tested) the cross-interference between TRP and KA.

Importantly, the cross-signal contribution could be considered non-significant for interferences II, III, V, and VI as compounds were separated chromatographically. The retention times for KYN and TRP-D_5_ were 1.75 and 2.25 min, while for TRP, XA, and KA, the retention times were 2.3, 6.15, and 6.1 min (Table 2). In the final method, despite using the scheduled MRM mode, interferences II and VI were present in the chromatograms as the retention times of KYN and TRP-D_5_ were close (e.g., see Figure S1B for interference II). This was not the case for interferences III and V, which were absent due to the set time windows for specific MRMs and the much different retention times of the analytes (compare Figure S1H).

### 3.2. Method Development and Validation

TRP is an essential, i.e., exogenous, amino acid [1,2]. Still, from the bioanalytical point of view, it needs to be treated as an endogenous molecule as it is detected in human matrices both in health ([19,22,30,31]) and disease ([11,14,15,16,18,21]). Different strategies have been described for endogenous compounds to overcome the lack of blank matrices in LC-MS/MS, one of which is using a “surrogate matrix” [32]. The developed method utilized human charcoal-stripped serum to prepare calibrators and quality control samples (QCs): we demonstrated that 300 mg/mL of activated charcoal sufficiently removed TRP and its major metabolites from human serum. The residual signals did not exceed 20% of the signal at the LLOQ level (Table S1 and Figure S1), and the matrix effects were acceptable (Table 3), which confirmed the suitability of charcoal-stripped serum as a surrogate matrix in the developed assay.

Other researchers also used pure solvents [13,14,17,19,22], charcoal-stripped BSA in PBS (bovine serum albumin in phosphate-buffered saline) [10], or BSA solution [21] for calibration curves in their TRP mass spectrometric assays. Although BSA is often utilized as a surrogate matrix in LC-MS/MS [32], it is not an ideal replacement in the TRP method as it contains TRP and its metabolites. Our preliminary experiments showed that 5% BSA in PBS contained amounts of TRP, KYN, KA, and AA that would affect the method’s LLOQs. On the other hand, pure solvent (PBS) generated unacceptable matrix effects for most analytes. Thus, we decided to use the charcoal-stripped serum to prepare calibrators and QCs as a matrix most similar to the authentic matrix. Adding a “W-shape” LC gradient at the end of each sample run (see “Materials and Methods”, Section 4.3) ultimately eliminated the matrix effect, which was initially recorded, most likely due to the late-eluting serum components, even when charcoal-stripped serum was used for calibration curves. We used a similar strategy in another LC-MS/MS method to eliminate persistent carry-over [33].

**Figure 4:**
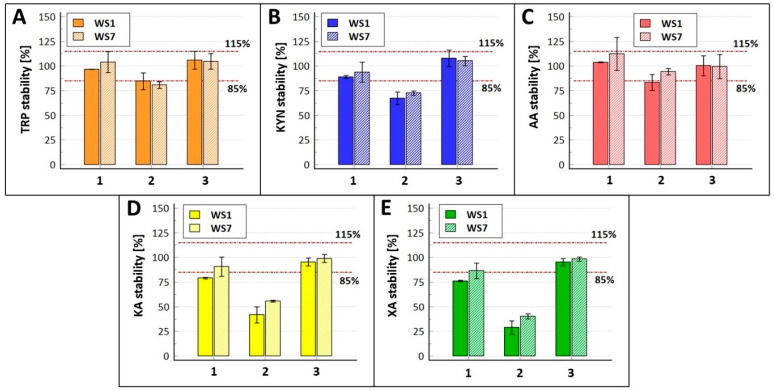
The stability of tryptophan and its metabolites in aqueous working solutions. The figure presents data for (**A**) tryptophan, (**B**) kynurenine, (**C**) anthranilic acid, (**D**) kynurenic acid, and (**E**) xanthurenic acid. The following conditions were assessed: (1) 1 h of stability at 20 °C, (2) 4 h of stability at 20 °C, and (3) 1 h of stability in a cooling rack. Stability was assessed at low and high concentrations (working solutions WS7 and WS1, respectively). The analyte/corresponding internal standard peak area ratio obtained for working solutions subjected to stress conditions was compared with fresh working solutions injected during the same analytical run. The experiment was conducted early during method development; unsatisfactory results forced a change in the solution solvent. The data are presented as means (*n* = 3, bars) ± STD (whiskers). The acceptable ranges (i.e., 85–115% of the initial values) are highlighted with red horizontal lines.

We initially observed a significant degradation of analytes when aqueous instead of methanolic working solutions were used (Figure 4). For KA and XA, even 1 h of stability at room temperature was unacceptable, and a cooling rack had to be used to avoid excessive degradation. This observation was surprising as some authors (e.g., [22,30]) used aqueous solutions in their TRP assays, and no degradation was reported during regular sample handling (which we attribute to a lack of this specific stability test). Finally, we used methanolic solutions, which provided sufficient stability for several hours at room temperature (Table S3), corroborating the literature [15]. Acidifying water or water-containing solvents (with citric, ascorbic, or formic acids) was proposed by others (e.g., [13,16,17]). Worse stability for XA compared to other TRP metabolites has also been reported for human plasma with various anticoagulants [14,22].

### 3.3. Study Strengths and Limitations

The proposed method is relatively fast and inexpensive. As the method will be used to assess the TRP metabolism in a large cohort of patients, we aimed to develop a method that was as cheap and straightforward as possible. Simple protein precipitation as a cleaning technique fully supports this goal. In contrast, some authors utilized solid-phase extraction (e.g., [10,13,34]), which is relatively expensive and, without automation, tedious and time-consuming. Moreover, other methods usually require a separate labeled internal standard (IS) for each analyte (Table 1), which would significantly increase the overall cost of the assay. We showed that implementing two ISs (3-NT for KYN and TRP-D_5_ for other compounds) led to acceptable accuracy, precision, and matrix effects (Tables S2 and S3). The KYN’s structural analog, 3-NT, was previously utilized as an IS for kynurenines by Sadok et al. [11], who showed that 3-NT behaved in the ion source similarly to KYN (no ion suppression) but not TRP, KA, and XA (for which signal suppression was recorded). These data support our observations that KYN needed a different IS than TRP, KA, and XA. The advantage of our method is also the ability to analyze TRP and its major metabolites in one analytical run, which was not always the case in previously published papers [10,11,14,15]. To decrease the overall signal response for TRP (and prevent mass spectrometer saturation), we applied lower collision energy than initially optimized (−5 V instead of −18 V). Other authors chose the isotopic ion ^13^C (M + 1) for TRP quantitation [19,22] and TRP product ions with lower signal intensity [22].

Moreover, we showed for the first time that charcoal stripping may have removed some substances from the serum that stabilized XA. In fact, the analyte was more unstable in the charcoal-stripped serum compared to the unstripped serum during multiple freezing and thawing (Table S3). This discrepancy highlights the importance of performing stability tests in the authentic matrix, even for the endogenous compounds, for which no truly blank matrix is available.

More importantly, our study was the first one to perform a separate cross-signal contribution experiment for TRP and its metabolites. According to our knowledge, no previous study included (or mentioned) this experiment in the validation report. Few mentioned cross-interferences [11,13,18] were noticed, most likely due to the different retention times of the analyzed compounds. We showed that injecting a high concentration of a single-compound solution and monitoring all other compounds’ MRM channels should be an integral part of method development. The procedure may reveal standard impurities (possible in the case of structurally related compounds), which would not be discovered if standards were injected only in a multi-analyte mixture (as in the calibrators). Moreover, the experiment can reveal cross-talk between the analyzed compounds. As we showed with TRP, KA, and XA, the cross-signal contribution may be present even if not suspected (due to different MRMs). In such cases, chromatographic separation is needed to ensure accurate results.

The presented method also has some limitations. Firstly, the method was validated only for four metabolites. In contrast, many recent methods allow for the determination of at least six metabolites (e.g., [11,12,13,16,19,22]). We also planned on including picolinic acid, 3-hydroxykynurenine, 3-hydroxyanthranilic acid, and quinolinic acid. However, the mass spectrometer signal for those analytes was unstable, and we failed to demonstrate the method’s robustness and repeatability. Thus, those TRP metabolites were excluded from the final validation.

Secondly, half of the samples from our cohort presented XA concentrations below the LLOQ (i.e., 8 nM, Figure 2E). This might suggest that serum XA concentrations in patients with cancer are lower than those in healthy people. In fact, the data in the literature for mean or medium blood XA (serum or plasma) ranges from around 14 to 30 nM in a non-cancer population [13,16,19,22,31]. Our stability tests, performed on pooled serum from healthy volunteers, also showed higher XA concentrations (~30 nM; Table S3). However, considering our previous efforts to increase the method’s sensitivity, we left the established LLOQ as it was. The planned biomarker study will further verify the method’s usefulness in determining XA concentrations in patients with lung cancer.

## 4. Materials and Methods

### 4.1. Chemicals and Reagents

Tryptophan (TRP, ≥98%, Cat. No. T0254), kynurenine (KYN, ≥98%, Cat. No. K8625), kynurenic acid (KA, ≥98%, Cat. No. K3375), xanthurenic acid (XA, 96%, D120804), anthranilic acid (AA, ≥98%, Cat. No. A89855), and 3-nitrotyrosine (3-NT, Cat. No. N7389) were provided by Merck/Sigma-Aldrich (Darmstadt, Germany). Tryptophan-D_5_ (TRP-D_5_, isotopic purity >95%, Cat. No. T947212) was obtained from Toronto Research Chemicals (North York, Kanada). All reference standards were stored according to the instructions provided by vendors. Formic acid (FA, LC-MS grade, 99%) was purchased from Carlo Erba (Val de Reuil, France), while activated carbon (Norit SX2) was purchased from Chempur (Piekary Śląskie, Poland). All LC-MS-grade solvents (water, methanol [MeOH], and acetonitrile [ACN]) were obtained from Merck (Darmstadt, Germany). Human serum for method validation was bought from the Regional Blood Donation and Blood Treatment Center (Poznań, Poland).

### 4.2. Stock and Working Solutions

Stock solutions were prepared by dissolving standards in 96% MeOH (for TRP and KYN) or 50% MeOH (for AA, KA, and XA) to obtain the final concentrations of 2.5 mM for TRP and KYN and 1.0 mM for AA, KA, and XA. The combined working solutions for TRP and metabolites were prepared in MeOH and had the following concentrations: 20–1250 µM for TRP, 1.6–100 µM for KYN, and 0.08–5 µM for KA, AA, and XA. Working solutions were aliquoted to 0.6 mL Eppendorf tubes and stored at −80 °C.

Two internal standards (ISs) were used for quantitation: TRP-D_5_ and 3-NT. Stock solution of TRP-D_5_ (5 mM) was prepared in MeOH, while 3-NT (0.5 mM) was prepared in 50% MeOH. The combined IS working solution (TRP-D_5_, 500 µM; 3-NT, 62.5 µM) was prepared in MeOH and was stored at −80 °C in aliquots.

### 4.3. Instrumentation and LC-MS/MS Conditions

The analysis was performed with a Shimadzu HPLC system coupled with an LCMS-8030 mass spectrometer (Shimadzu, Kyoto, Japan). The instrument was operated in positive ion mode (ESI+) with the following parameters: a desolvation line temperature of 240 °C; a heat block temperature of 350 °C; a needle voltage of 4.5 kV; nebulizing gas at a rate of 2 L/min; and drying gas at a rate of 12 L/min. Nitrogen was used as nebulizing and drying gas, while argon was used as collision-induced dissociation gas. In the final LC-MS/MS method, a scheduled MRM strategy was applied. Table 4 presents the MRM transitions used for quantitation (along with their settings), as well as the molecular weights and the retention times of the analytes and their ISs.

**Figure 5:**
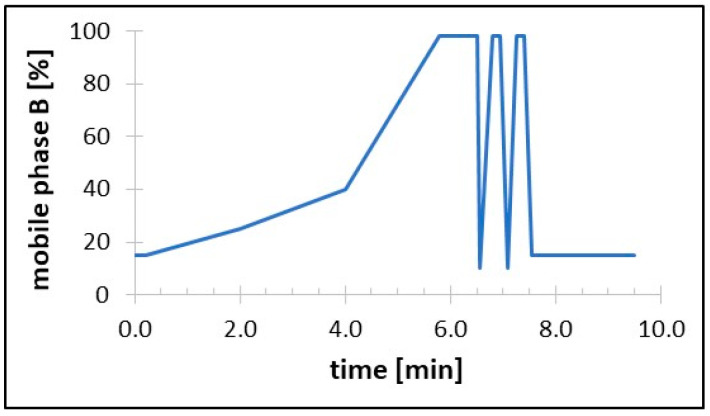
The LC gradient profile utilized in the method. The following gradient profiles were implemented at a flow rate of 0.4 mL/min: 0.00–0.20 min, 15% MeOH; 0.20–2.00 min, 15–25% MeOH; 2.00–4.00 min, 25–40% MeOH; 4.00–5.80 min, 40–98% MeOH; 5.80–6.50 min, 98% MeOH; 6.50–6.55 min, 98–10% MeOH; 6.55–6.80, 10–98% MeOH; 6.80–6.95 min, 98% MeOH; 6.95–7.10 min, 98–10% MeOH; 7.10–7.25, 10–98% MeOH; 7.25–7.40 min, 98% MeOH; 7.40–7.55, 98–15% MeOH; and 7.55–9.50 min, 15% MeOH. The mobile phase was directed to waste for the first 1.0 min and the last 2.0 min of the assay.

The compounds were separated on a Synergi Polar-RP 80 column (100 × 2 mm, 4 μm; Phenomenex, Torrance, CA, USA) preceded by the corresponding guard column. A mixture of water and 0.1% FA was used as mobile phase A, and MeOH was used as mobile phase B. Figure 5 shows the LC gradient used to separate the analytes. The autosampler needle was washed externally before and after aspiration using MeOH and water (1:1, *v*/*v*). The column and autosampler temperatures were 25 °C and 10 °C, respectively. An amount of 10 µL was injected into the column.

### 4.4. Surrogate Matrix

Charcoal-stripped serum was used as a surrogate matrix for calibrators and QCs. It was obtained by shaking human serum with activated charcoal (300 mg/mL; 6 h, 4 °C, 750 rpm), and then double centrifugation (10 and 5 min; 4 °C; 40,930× *g*) and filtering (5 min; 4 °C; 10,000× *g*) through 0.45 µm Costar Spin-X centrifugal filters with cellulose acetate membrane (Cat. No. 8163). A surrogate matrix was stored at −80 °C until used.

### 4.5. Sample Preparation

An amount of 75 µL of charcoal-stripped serum was mixed well with 7.5 µL of working solutions and 10 µL of IS (TRP-D_5_, 500 µM; 3-NT, 62.5 µM) to prepare calibrators and QCs. For study samples, 75 µL of the patient’s serum was spiked with 7.5 µL of MeOH and 10 µL of IS. The proteins were then precipitated by adding 300 µL of ice-cold (−20 °C) mixture of ACN and MeOH (1:1, *v*/*v*), shaking for 10 min (4 °C, 750 rpm), and centrifuging for another 10 min (4 °C, 40,930× *g*). The supernatant was evaporated to dryness under reduced pressure using Eppendorf Concentrator Plus (room temperature, V-AQ mode). The dry residue was re-dissolved in a mixture of 0.1% FA in water and MeOH (80:20, *v*/*v*; 50 µL) and centrifuged for 5 min (4 °C, 40,930× *g*). The supernatant was transferred to HPLC vials with conical inserts and injected into the column.

### 4.6. Cross-Signal Contribution Experiment

Due to the similar structures of the analyzed compounds (Figure 1), a cross-signal contribution experiment was performed during method development. Cross-signal contribution was defined as an unexpected response in the ion chromatogram of a specific compound after injecting another compound. The experiment involved injecting a solution of a pure compound and monitoring the MRM channels of all other analyzed compounds. The analytes (i.e., TRP, KYN, AA, KA, and XA) were injected at the concentrations that reflected the method’s upper limit of quantitation (ULOQ), while the ISs (3-NT and TRP-D_5_) were injected at concentrations mimicking the ones used in the study samples. The peak areas and retention times were recorded for all monitored MRM channels. The scheduled MRM mode was turned off during the cross-signal contribution experiment to reveal all potential cross-interferences.

For interferences that matched the compound’s retention time, the experiment was repeated with the charcoal-stripped serum to assess the extent of interference during the analytical batch. The peak areas of all interferences recorded at the retention time of the analyte had to be ≤20% of the analyte’s peak area at the LLOQ level and for IS ≤ 5% to meet the validation guidelines.

### 4.7. Method Validation

The TRP method was validated according to the International Council for Harmonization (ICH) guidelines [20]. The following parameters were evaluated: selectivity, linearity, accuracy, precision, matrix effect, carry-over, and stability. Moreover, the feasibility of using charcoal-stripped serum as a “surrogate matrix” was assessed. A detailed description of the experiments and the acceptance criteria are presented below.

#### 4.7.1. Selectivity

Selectivity was assessed by inspecting chromatograms of blank samples (i.e., samples with no analytes and no IS) prepared from ten different LOTs of charcoal-stripped serum. The peak areas detected at the retention times of monitored compounds could not exceed 20% of the analytes’ peak areas and 5% of the IS areas in the samples at the LLOQ level injected during the same run.

#### 4.7.2. Linearity

Calibration curves were constructed by plotting the peak area ratios (analyte/appropriate IS) versus the nominal concentrations. For each analyte, two regression models (linear regression with 1/x^2^ weighting and power function) and two ISs (TRP-D_5_ and 3-NT) were tested, and their performance was evaluated based on the results obtained from the accuracy and matrix effect experiments.

#### 4.7.3. Accuracy and Precision

Intra-day accuracy and precision were assessed by preparing four sets of QCs (*n* = 5) covering the method’s LLOQ, low (LQC), medium (MQC), and high concentrations (HQC). Inter-day accuracy and precision were assessed by analyzing LLOQ, LQC, MQC, and HQC samples over several days (*n* = 5). Accuracy was expressed as a percentage of the nominal concentration, while precision was expressed as a percent coefficient of variation (%CV). Results within 85–115% (80–120% for the LLOQ) for accuracy and ≤15% (≤20% for the LLOQ) for precision were considered acceptable.

#### 4.7.4. Matrix Effect

The matrix effect was assessed at low and high concentrations for serum from six subjects. For each concentration level, two sets of samples were prepared. Series A (*n* = 2) reflected the endogenous levels of the analytes (samples were prepared in the same way as the study samples; see Section 4.5). Series B (*n* = 3) contained serum spiked with the appropriate working solution (instead of pure MeOH, samples were spiked with working solution WS_2_ at high concentrations or WS_6_ at low concentrations). The resulting concentrations were read from the calibration curves prepared in charcoal-stripped serum. The matrix effect was calculated as a percentage of the expected concentration with the equation ME[%] = B/(A + WS_2/6_) × 100%, where A corresponds to the mean concentration from series A, B corresponds to the concentrations from series B (matrix effect was calculated separately for each of three samples), and WS_2/6_ corresponds to the QC level appropriate for WS_2_ or WS_6_, respectively. Mean ME within 85–115% and CV ≤ 15% for each individual serum were considered acceptable.

#### 4.7.5. Carry-Over

Carry-over was assessed by repeatedly injecting the HQC sample (*n* = 5), equal to the method’s ULOQs, followed by the sample solvent (0.1% FA in water/MeOH, 80:20, *v*/*v*). The observed peak areas in the blank solvent could not exceed 20% of the analyte’s peak areas and 5% of the IS’s peak area registered for the LLOQ samples injected during the same run (*n* = 5). The reconstitution solvent rather than blank samples was used to assess carry-over as charcoal stripping was not always 100% efficient (Table S1), and interferences in blank samples could have adversely affected the experiment results.

#### 4.7.6. Stability

Stability in the matrix was assessed at two concentration levels (LLOQ and HQC) by preparing sets of QC samples (*n* = 3) in charcoal-stripped serum. In order to mimic study sample handling, all QCs were frozen for at least 12 h at −80 °C before being analyzed against a fresh calibration curve. The following conditions were assessed: 1 h of stability at room temperature, 5 weeks of stability at −80 °C, stability after three freeze–thaw cycles (from −80 °C to room temperature), and 6 h of stability in the autosampler. The mean back-calculated concentrations within 85–115% for HQC and 80–120% for LLOQ were considered acceptable.

The stability in serum was also confirmed by analyzing pooled human serum subjected to multiple cycles of freezing and thawing or left for 2 h on the benchtop or in the autosampler for 6 h. The results of these tests were considered acceptable if the back-calculated concentrations were within 85–115% of the initial concentrations determined after the 1st freeze–thaw cycle (these samples were processed immediately after thawing without unnecessary delay). Unfortunately, we did not have fresh (unfrozen) human serum for the additional stability tests. However, all study samples in the planned biomarker study would also be analyzed after at least one cycle of freezing and thawing. Thus, the experiments assessed whether regular sample handling (like keeping them on the benchtop or in the autosampler) or additional freeze–thaw cycles would adversely affect determined concentrations.

The stability of analytes in working solutions was assessed for the lowest (WS_7_) and the highest (WS_1_) concentrations by evaporating to dryness (*n* = 3) a mixture of 7.5 µL of the appropriate working solution and 10 µL of the IS and reconstituting in 50 µL of the sample diluent (0.1% FA in water and MeOH, 80:20, *v*/*v*). The peak area ratio of analyte/IS obtained for working solutions subjected to stress conditions was compared with fresh working solutions injected during the same analytical run. The following conditions were assessed: 1–4 h of stability at room temperature (~20 °C), 5 weeks of stability at −80 °C, and stability after four cycles of freezing and thawing (from −80 °C to room temperature). Mean results within 80–120% for WS_7_ and 85–115% for WS_1_ were considered acceptable.

### 4.8. Clinical Application

The method was used to determine the concentrations of TRP and its metabolites in 20 patients (10 men and 10 women) with advanced lung cancer undergoing chemoimmunotherapy with pembrolizumab. The median age of participants was 68 years, and the median BMI was 26.2 kg/m^2^. Serum samples were collected just before the first dose of pembrolizumab (*n* = 20) and then repeatedly during the treatment (pre-dose, before the second, third, or fourth dose of the drug; *n* = 51) and immediately stored at −80 °C in aliquots. This study was approved by the Bioethics Committee at Poznan University of Medical Sciences (decision No. 80/19 and 562/22).

## 5. Conclusions

A robust and straightforward LC-MS/MS method has been developed and successfully validated. This method can be used to assess TRP metabolism in patients with lung cancer. Various observed cross-interferences highlight the need to test for cross-signal contribution during method development and to pay more attention to analyte resolution in the case of mass spectrometric assays. The demand for high-throughput methods and the favorable dilute-and-shoot approach may trigger neglect of the time-consuming and sophisticated chromatography. However, it is crucial to keep in mind that the LC part is as important as the MS/MS part in the LC-MS/MS methods and that only their perfect cooperation guarantees reliable quantification.

## Figures and Tables

**Table 2:** Detailed information on the detected interferences.

		Injected Compound (Pure Single-Compound Solution) ^a^						
		TRP (125 µM)	KYN (10 µM)	AA (500 nM)	KA (500 nM)	XA (500 nM)	3-NT (~8.5 µM)	TRP-D_5_ (~67 µM)
Monitored MRM channel (Q1 > Q3, *m*/*z*)	**TRP** (204.8 > 145.9)	+ [2.3 min]	-	-	-	-	-	-
	**KYN** (208.8 > 191.9)	+ ^b^ [1.75 min]	+ [1.75 min]	-	-	-	-	+ ^c^ [2.25 min]
	**AA** (137.8 > 120.0)	-	-	+ [4.3 min]	-	-	-	-
	**KA** (189.8 > 144.0)	+ ^d^ [2.3 min]	+ ^e^ [6.1 min]	-	+ [6.1 min]	-	-	-
	**XA** (206.0 > 159.9)	+ ^f^ [2.3 min]	-	-	-	+ [6.15 min]	-	-
	**3-NT** (227.0 > 180.9)	-	-	-	-	-	+ [1.7 min]	-
	**TRP-D_5_** (209.8 > 192.0)	-	+ ^g^ [1.75 min]	-	-	-	-	+ [2.25 min]

^a^ Analytes (TRP, KYN, AA, KA, and XA) were injected at the ULOQ level, while ISs (3-NT and TRP-D_5_) were injected at the concentrations used in the study samples (“+” means that a peak was detected in the specific MRM channel at the retention time provided in parenthesis; “-“ means that no peak was recorded in the specific MRM channel; and the expected peaks are marked in gray); ^b^ **interference I** (~0.7% of the KYN’s peak area at the ULOQ level; the experiment repeated in charcoal-stripped serum confirmed that the interference did not exceed 20% of the KYN’s peak area at the LLOQ level); ^c^ **interference II** (~72% of the KYN’s peak area at the ULOQ level); ^d^ **interference III** (~1.3% of the KA’s peak area at the ULOQ level); ^e^ **interference IV** (~1.5% of the KA’s peak area at the ULOQ level; the experiment repeated in charcoal-stripped serum confirmed that the interference did not exceed 20% of the KA’s peak area at the LLOQ level); ^f^ **interference V** (~285% of the XA’s peak area at the ULOQ level); ^g^ **interference VI** (~0.8% of the TRP-D_5_’s peak area in the calibrators and QC samples). *Abbreviations:* 3-NT, 3-nitrotyrosine; AA, anthranilic acid; IS, internal standard; KA, kynurenic acid; KYN, kynurenine; LLOQ, lower limit of quantitation; MRM, multiple reaction monitoring; QC, quality control; TRP, tryptophan; TRP-D_5_, deuterated tryptophan; ULOQ, upper limit of quantitation; XA, xanthurenic acid.

**Table 3:** Mean matrix effects in human serum for six individual subjects.

	TRP	KYN	AA	KA	XA
Low concentrations ^a^	100.2–107.4 [1.0–5.4]	101.8–110.4 [1.0–5.6]	95.5–110.0 [0.8–10.3]	95.4–105.1 [1.2–5.1]	85.6–96.7 [0.9–8.6]
High concentrations ^a^	97.3–106.3 [1.1–4.9]	98.1–106.8 [0.4–4.0]	98.1–109.5 [1.3–4.5]	88.5–109.2 [0.6–3.5]	87.8–103.9 [2.4–7.1]
Total ME ^b^	102.3 ± 3.3 [3.3]	104.5 ± 3.3 [3.2]	103.8 ± 5.4 [5.2]	99.2 ± 5.8 [5.9]	93.3 ± 4.9 [5.3]

^a^ Results are presented as ranges of mean (*n* = 3) MEs and their %CVs calculated for individual matrices (*n* = 6); ^b^ mean ME ± STD [%CV] observed across both concentrations (low and high) for all tested LOTs of matrix.

**Table 4:** The compound-dependent LC-MS/MS parameters.

	MW [g/mol]	Q1 [*m*/*z*]	Q3 [*m*/*z*]	Q1 Pre Bias [V]	CE [V]	Q3 Pre Bias [V]	RT [min]	MRM Window [min]
TRP	204.22	204.80	145.90	−30.0	−5.0	−26.0	2.30	1.8–3.2
KYN	208.21	208.80	191.90	−30.0	−10.0	−20.0	1.75	1.3–3.0
AA	137.14	137.85	120.00	−30.0	−15.0	−21.0	4.30	3.6–5.4
KA	189.17	189.75	144.00	−30.0	−20.0	−25.0	6.10	5.3–7.2
XA	205.17	206.00	159.90	−27.0	−20.0	−29.0	6.15	5.3–7.2
3-NT ^a^	226.19	227.00	180.90	−12.0	−15.0	−18.0	1.70	1.3–2.4
TRP-D_5_ ^b^	209.26	209.80	191.95	−30.0	−10.0	−20.0	2.25	1.8–3.2

^a^ internal standard for KYN; ^b^ internal standard for TRP, AA, KA, and XA. *Abbreviations*: 3-NT, 3-nitrotyrosine; AA, anthranilic acid; CE, collision energy; KA, kynurenic acid; KYN, kynurenine; MRM, multiple reaction monitoring; MW, molecular weight; *m*/*z*, mass-to-charge ratio; Q1 and Q3, quadrupole 1 and quadrupole 3; RT, retention time; TRP, tryptophan; TRP-D_5_, tryptophan-D_5_; XA, xanthurenic acid.

## Data Availability

All relevant data are included in this article and presented as figures, tables, or Supplementary Materials. Other data are available from the corresponding author upon reasonable request.

## Supplementary Materials

The following supporting information can be downloaded at https://www.mdpi.com/article/10.3390/molecules30010121/s1, Table S1: The results from the selectivity experiment; Table S2: Calibration ranges and mean intra-day and inter-day accuracy and precision in charcoal-stripped serum; Table S3: Stability for tryptophan and its metabolites under various storage conditions; Figure S1: Exemplary ion chromatograms for the developed LC-MS/MS method; Figure S2: Exemplary calibration curves for (A) tryptophan, (B) kynurenine, (C) anthranilic acid, (D) kynurenic acid, and (E) xanthurenic acid; Figure S3: Cross-signal contribution between kynurenine (KYN) and deuterated tryptophan (TRP-D_5_) in all monitored MRM channels; Figure S4: The product ion scan for tryptophan-D_5_ (precursor ion of 209.8 *m*/*z*).
