# Supplementary material for: A Novel Liquid Chromatography–Tandem Mass Spectrometry Method to Quantify Tryptophan and Its Major Metabolites in Serum to Support Biomarker Studies in Patients with Cancer Undergoing Immunotherapy
Source: Molecules. 2024 Dec 31;30(1):121. doi: 10.3390/molecules30010121 (PMC11721486; doi:10.3390/molecules30010121)
Supplement: Supplementary file 1 [file molecules-30-00121-s001.zip › molecules-3350741-supplementary.pdf]

Supplementary Materials

A Novel Liquid Chromatography–Tandem Mass Spectrometry Method to Quantify Tryptophan and Its Major Metabolites in Serum to Support Biomarker Studies in Patients with Cancer Undergoing Immunotherapy

Anna Siemiątkowska *, **Barbara Kuźnar-Kamińska** #, **Katarzyna Kosicka-Noworzyń** #, **Kamila Nowaczewska, Hanna Winiarska, Dominika Popiołek, Filip Kamiński, Franciszek K. Główka**

*corresponding author (asiemiatkowska@ump.edu.pl)

# These authors contributed equally to this work.

TABLES and FIGURES:

Table S1. The results from the selectivity experiment

Table S2. Calibration ranges and mean intra-day and inter-day accuracy and precision in charcoal-stripped serum

Table S3. Stability for tryptophan and its metabolites under various storage conditions

Figure S1. Exemplary ion chromatograms for the developed LC-MS/MS method.

Figure S2. Exemplary calibration curves for (**A**) tryptophan, (**B**) kynurenine, (**C**) anthranilic acid, (**D**) kynurenic acid, and (**E**) xanthurenic acid.

Figure S3. Cross-signal contribution between kynurenine (KYN) and deuterated tryptophan (TRP-D_5) in all monitored MRM channels.

Figure S4. The product ion scan for tryptophan-D_5 (precursor ion of 209.8 *m*/*z*).

Table S1. The results from the selectivity experiment

Analyte	% of the LLOQ's peak area	
	Median (range)	Mean ± STD
**Tryptophan (TRP)**	1.8 (0.0 – 5.9)	2.1 ± 1.6
**Kynurenine (KYN)**	0.0 (0.0 – 5.8)	1.0 ± 1.9
**Anthranilic acid (AA)**	0.0 (0.0 – 0.0)	0.0 ± 0.0
**Kynurenic acid (KA)**	0.0 (0.0 – 0.0)	0.0 ± 0.0
**Xanthurenic acid (XA)**	0.0 (0.0 – 7.2)	1.2 ± 2.6
**3-nitrotyrosine (3-NT, IS)**	0.0 (0.0 – 0.0)	0.0 ± 0.0
**Tryptophan-D_5 (TRP-D_5, IS)**	0.0 (0.0 – 0.0)	0.0 ± 0.0

The experiment assessed the method's selectivity and efficacy in removing endogenous TRP and its metabolites from human serum with 300 mg/mL of activated charcoal. It was performed by processing 10 independent blank samples (charcoal-stripped serum unspiked neither with the analytes nor the ISs) and comparing the peak areas in blank samples with those registered at the lower limit of quantitation level (LLOQ) injected during the same analytical run. The results were expressed in %.

Table S2. Calibration ranges and mean intra-day and inter-day accuracy and precision in charcoal-stripped serum

Analyte	IS	Calibration range	Accuracy and precision [a]			
			Intra-day (*n* = 5)		Inter-day (*n* = 5)	
			LLOQ	other QCs [b]	LLOQ	other QCs [b]
**TRP**	TRP-D_5	2 – 125 µM	95.1 [3.7]	96.9 – 99.4 [2.4 – 3.6]	99.3 [2.0]	95.0 – 103.8 [1.9 – 2.5]
**KYN**	3-NT	0.16 – 10 µM	97.5 [2.8]	96.6 – 105.5 [2.6 – 3.6]	98.7 [2.9]	94.7 – 103.5 [1.8 – 2.7]
**AA**	TRP-D_5	8 – 500 nM	91.8 [8.9]	95.9 – 102.0 [2.7 – 5.2]	106.7 [4.8]	94.1 – 107.5 [2.1 – 2.9]
**KA**	TRP-D_5	8 – 500 nM	94.4 [1.8]	93.0 – 98.4 [2.7 – 6.3]	103.4 [3.9]	94.3 – 107.0 [2.4 – 3.1]
**XA**	TRP-D_5	8 – 500 nM	101.2 [8.5]	95.7 – 104.6 [3.9 – 5.4]	103.0 [3.2]	96.5 – 104.6 [3.6 – 4.5]

[a] Results are presented as a % of the nominal concentration [%CV]; [b] LQC, MQC, HQC;

LLOQ was 2 µM for TRP, 0.16 µM for KYN, and 8 nM for AA, KA, and XA;
LQC was 5 µM for TRP, 0.40 µM for KYN, and 20 nM for AA, KA, and XA;
MQC was 50 µM for TRP, 4 µM for KYN, and 200 nM for AA, KA, and XA;
HQC was 125 µM for TRP, 10 µM for KYN, and 500 nM for AA, KA, and XA.

Abbreviations: 3-NT, 3-nitrotyrosine; AA, anthranilic acid; CV, coefficient of variation; HQC, QC at high concentration; IS, internal standard; KA, kynurenic acid; KYN, kynurenine; LLOQ, lower limit of quantitation; LQC, QC at low concentration; MQC, QC at medium concentration; QC, quality control sample; TRP, tryptophan; TRP-D_5, tryptophan-D_5; XA, xanthurenic acid.

Table S3. Stability for tryptophan and its metabolites under various storage conditions

		**TRP**	**KYN**	**AA**	**KA**	**XA**
		**methanolic working solutions [a]**				
**4 h at 20 °C**	low conc.	95.9 [8.1]	98.2 [11.3]	98.4 [14.5]	97.6 [11.5]	94.3 [3.8]
	high conc.	104.6 [7.5]	98.1 [7.6]	93.8 [11.6]	100.5 [6.1]	98.3 [5.4]
**4 FT cycles**	low conc.	91.6 [11.0]	99.2 [9.6]	87.4 [6.5]	90.9 [19.0]	91.8 [4.0]
**(−80 °C → 20 °C)**	high conc.	102.4 [4.8]	106.9 [4.4]	100.7 [3.5]	103.5 [5.6]	107.0 [6.2]
**5 weeks at −80 °C**	low conc.	97.3 [6.3]	101.1 [6.1]	102.5 [10.9]	98.1 [5.3]	95.2 [11.2]
	high conc.	107.5 [11.8]	110.7 [12.3]	109.3 [11.8]	102.0 [11.3]	113.0 [9.4]
		**charcoal-stripped serum [b]**				
**1 FT cycle**	low conc.	91.8 [5.3]	91.8 [7.2]	101.4 [3.0]	96.9 [7.4]	105.8 [9.2]
**+ 1 h at 20 °C**	high conc.	97.5 [0.7]	95.0 [1.7]	102.5 [2.0]	99.6 [0.9]	95.2 [1.3
**1 FT cycle**	low conc.	95.0 [4.0]	99.0 [4.9]	104.6 [3.7]	108.2 [5.1]	92.3 [5.1]
**+ 5 wks at −80 °C**	high conc.	94.8 [5.7]	104.3 [5.1]	103.3 [6.1]	99.6 [6.2]	92.3 [6.3]
**3 FT cycles**	low conc.	96.5 [2.4]	93.5 [1.5]	98.4 [12.7]	93.8 [8.1]	83.3 [11.1]
**(−80 °C → 20 °C)**	high conc.	91.9 [1.1]	93.8 [1.4]	94.1 [1.3]	91.3 [1.9]	85.9 [2.2]
**autosampler stab.**	low conc.	94.9 [1.0]	86.2 [3.1]	106.2 [1.9]	108.5 [5.2]	108.4 [6.3]
**(6 h at 10 °C)**	high conc.	99.0 [2.2]	100.6 [2.1]	114.3 [2.6]	110.9 [3.1]	113.3 [4.1]
		**pooled human serum [c]**				
**1 FT cycle + 2 h at 20 °C**		97.6 [0.8]	99.7 [1.4]	90.8 [6.6]	100.0 [1.3]	98.0 [6.8]
**3 FT cycles (−80 °C → 20 °C)**		98.9 [0.5]	101.1 [1.4]	92.8 [7.7]	102.1 [1.9]	105.3 [4.7]
**autosampler stab. (6 h at 10 °C)**		99.8 [2.0]	100.0 [1.2]	97.3 [8.4]	112.3 [3.3]	105.2 [2.3]

results are presented as mean [%CV], n = 3;

[a] stability was calculated by comparing the peak area ratios of analyte/IS for working solutions subjected to stress conditions and fresh working solutions injected during the same analytical run and was expressed in %; at low concentrations, stability was assessed for working solution WS_7, at high concentrations – WS_1;

[b] stability was assessed by preparing the quality control samples in charcoal-stripped serum and was calculated as a % of the nominal concentration; at low concentrations, serum was spiked with WS_7, at high concentrations – WS_1;

[c] stability was calculated as a % of the initial concentration; the mean initial concentrations (i.e., the concentrations determined after the 1st FT cycle) were as follows (n = 4): 85.18 µM for TRP, 2.19 µM for KYN, 8.88 nM for AA, 40.14 nM for KA, and 28.48 nM for XA.

Abbreviations: AA, anthranilic acid; CV, coefficient of variation; FT, freeze-thaw; KA, kynurenic acid; KYN, kynurenine; STD, standard deviation; TRP, tryptophan; XA, xanthurenic acid.

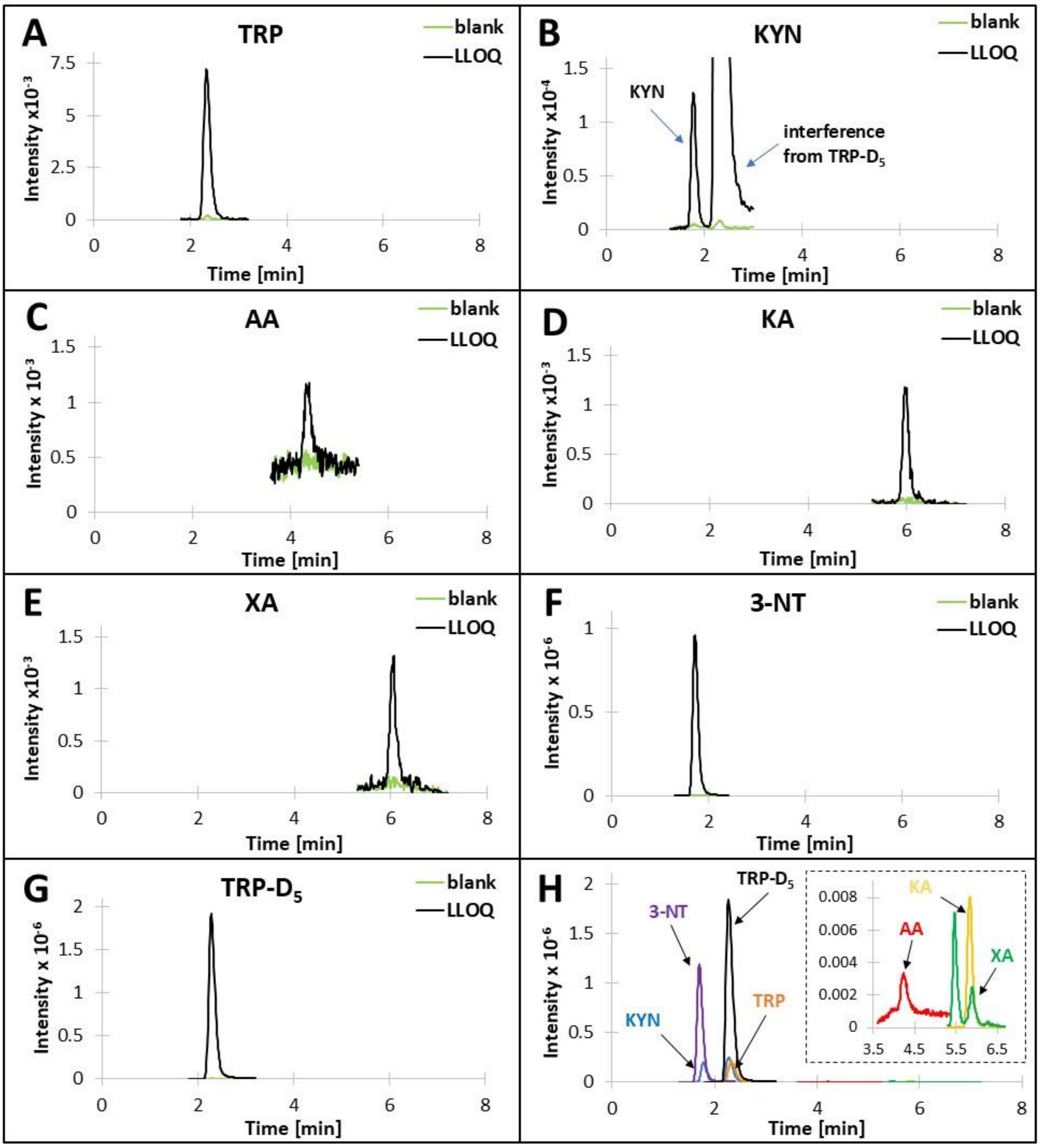

Figure S1. Exemplary ion chromatograms for the developed LC-MS/MS method.

(A – G) blank and LLOQ samples in charcoal-stripped serum [(**A**) presents the MRM channel for tryptophan, (**B**) kynurenine, (**C**) anthranilic acid, (**D**) kynurenic acid, (**E**) xanthurenic acid, (**F**) internal standard, 3-nitrotyrosine, (**G**) internal standard, deuterated tryptophan]; (**H)** serum of a lung cancer patient dosed with chemoimmunotherapy (determined concentrations: 61.00 µM for TRP, 3.22 µM for KYN, 25.37 nM for AA, 31.06 nM for KA, 8.62 nM for XA).

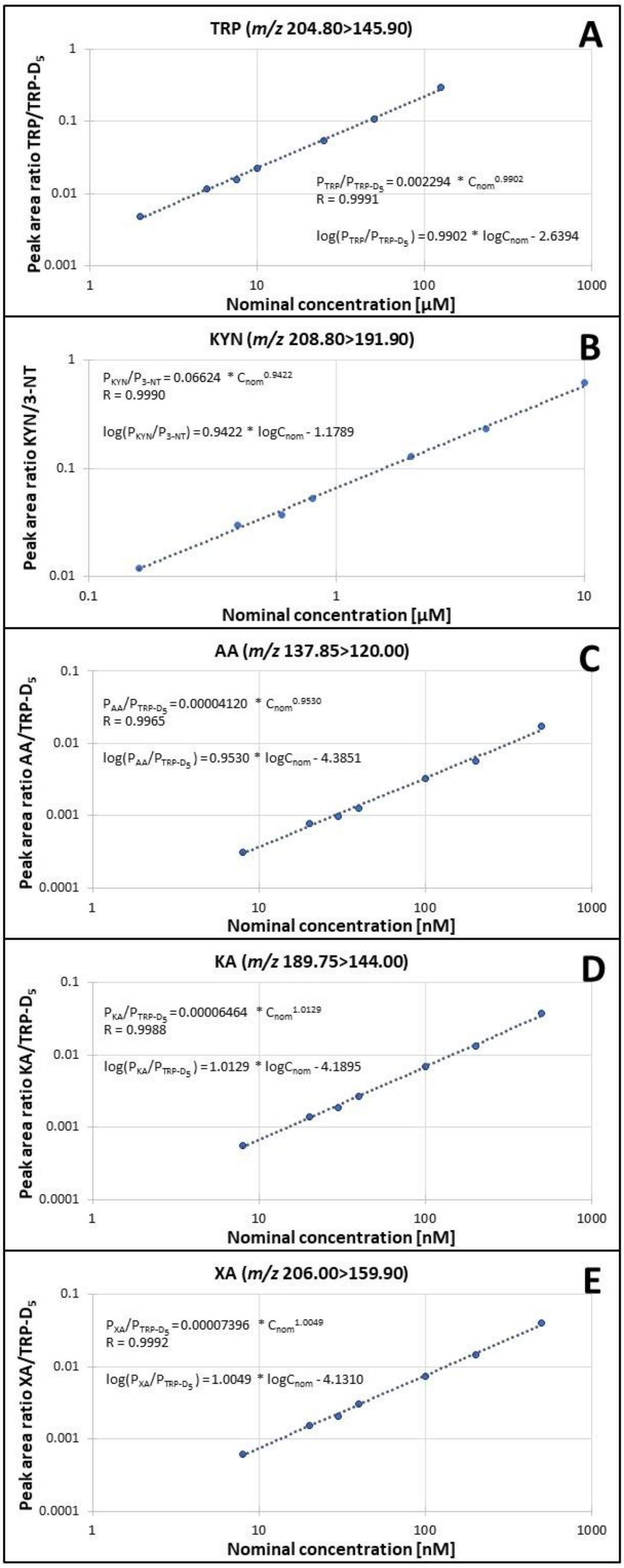

Figure S2. Exemplary calibration curves for (**A**) tryptophan, (**B**) kynurenine, (**C**) anthranilic acid, (**D**) kynurenic acid, and (**E**) xanthurenic acid.

Abbreviations: 3-NT, 3-nitrotyrosine (internal standard); C_{nom}, nominal concentration; *m*/*z*, mass-to-charge ratio; P, peak area; TRP-D_5, tryptophan-D_5 (internal standard).

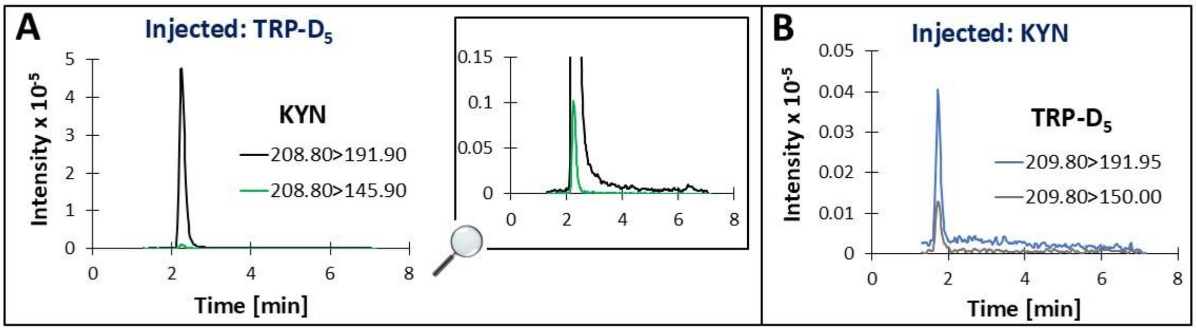

Figure S3. Cross-signal contribution between kynurenine (KYN) and deuterated tryptophan (TRP-D_5) in all monitored MRM channels.

(**A**) peaks recorded in the MRM channels of KYN (retention time of 1.75 min) after injection of a high concentration of TRP-D_5 (retention time of 2.25 min); (**B**) peaks recorded in the MRM channels of TRP-D_5 (retention time of 2.25 min) after injection of a high concentration of KYN (retention time of 1.75 min).

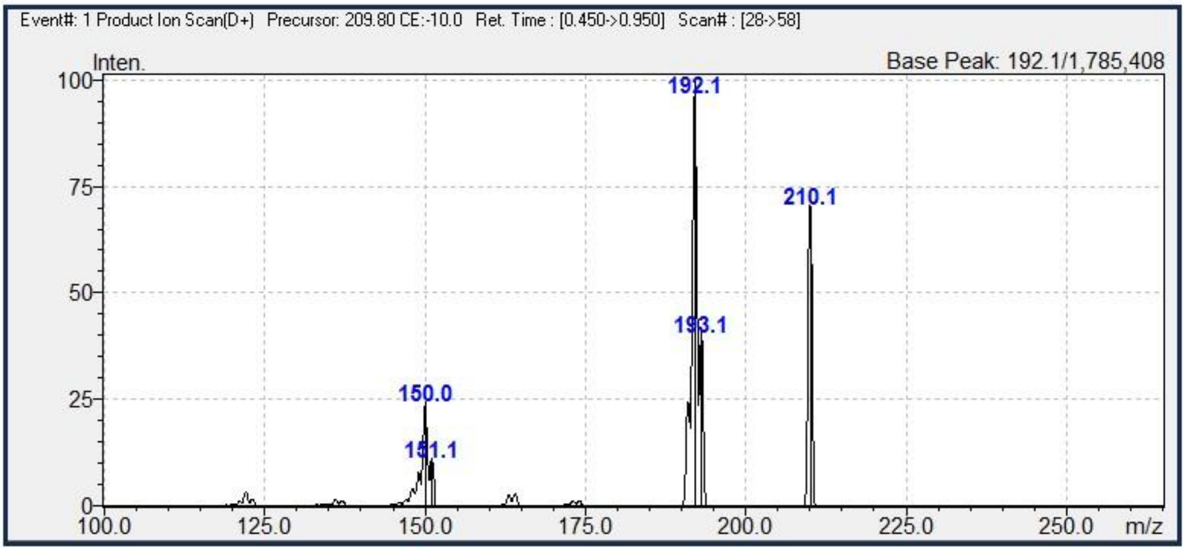

Figure S4. The product ion scan for tryptophan-D_5 (precursor ion of 209.8 *m*/*z*).
